# Diversity of bacterium communities in saline-alkali soil in arid regions of Northwest China

**DOI:** 10.1186/s12866-021-02424-7

**Published:** 2022-01-06

**Authors:** Lili Nan, Quanen Guo, Shiyu Cao, Zongbing Zhan

**Affiliations:** 1 College of Grassland Science, Key Laboratory of Grassland Ecosystem (Ministry of Education), Gansu Agricultural University, Lanzhou, 730070 Gansu China; 2 Institute of Soil, Fertilizer and Water-saving, Gansu Academy of Agricultural Sciences, Lanzhou, 730070 Gansu China

**Keywords:** Illumina MiSeq sequencing, Bacterial community, Driving force, Diversity, Saline-alkali soil

## Abstract

**Background:**

The saline-alkali soil area accounts for over 1/4-1/5 of the land area in Gansu Province of China, which are mainly distributed in the north of Hexi corridor and Jingtai basin. The unique ecological environment contains unique and diverse microbial resources. The investigation of microbial diversity in saline environment is vital to comprehend the biological mechanisms of saline adaption, develop and utilize microbial resources.

**Results:**

The Illumina MiSeq sequencing method was practiced to investigate the bacterial diversity and composition in the 5 subtypes and 13 genera of saline-alkali soil in Gansu Province, China. The results from this study show that *Proteobacteria*, *Bacteroidetes*, *Actinobacteria*, *Firmicutes*, and *Gemmatimonadetes* were the dominant bacterial groups in 13 saline soil. *Proteobacteria* had the greatest abundance in sulfate-type meadow solonchaks and orthic solonchaks, chloride-type orthic solonchaks and bog solonchaks, sulfate-chloride-type, chloride-sulfate-type, and sulfate-type dry solonchaks. *Halobacteria* was the dominant bacterial class in soil samples except for sulfate-type meadow solonchaks and orthic solonchaks, chloride-type orthic solonchaks and bog solonchaks. The richness estimators of Ace and Chao 1 and the diversity indices of Shannon and Simpson revealed the least diversity in bacterial community in sulfate-chloride-type orthic solonchaks.

**Conclusions:**

The sulfate anion was the most important driving force for bacterial composition (17.7%), and the second most influencing factor was pH value (11.7%).

## Background

Soil salinization has become a worldwide problem that menaces the growth and yield of crops and impedes the development of modern agricultural sustainability [20]. In Gansu Province of China, about 1.02 × 10^6^ hm^2^ of cropping land are affected by soil salinity or sodicity [15], the salinity is mainly of sulfate-type saline soil (37.9%) or chloride – sulfate – type saline soil (35.3%). High salt concentrations as well as an uneven temporal and spatial water distribution have a negative impact on sustainable development in agriculture. Soil microorganisms, playing a central role in soil organic matter decomposition, nutrient transformations, enzyme production, and soil quality maintenance [27, 35], can be greatly affected by the salt concentration. For example, moderately halophilic bacteria to extremely halophilic bacteria community can be found in hypersaline environment [21]. Microbes in saline soils varied greatly from the non-saline environment [4], and halophilic and halotolerant bacteria community are essential for the biogeochemical processes in hypersaline soil [24].

Archaea, fungi, bacteria, and viruses are the four major microbial taxa in soil [13]. Archaea can grow in conditions of extreme salinity, temperature and pH, in which bacteria cannot grow [36]. Saline-alkaline soils generally lack fungi [39], and fungi tend to be sensitive to salt stress [33], and long term salt stress reduces fungal diversity [3]. Bacteria are the most abundant microorganisms in the soil [31]. Their ecology, structure, diversity, and population dynamics are driven by soil physicochemical characteristics [12] such as soil organic carbon content [17], pH [11, 34], soil electrical conductivity [38], the plant secretion [32], trophic status [40], geographic distance [41], salinity [22], and types and concentrations of soil ions [19, 43].

**Fig. 1:**
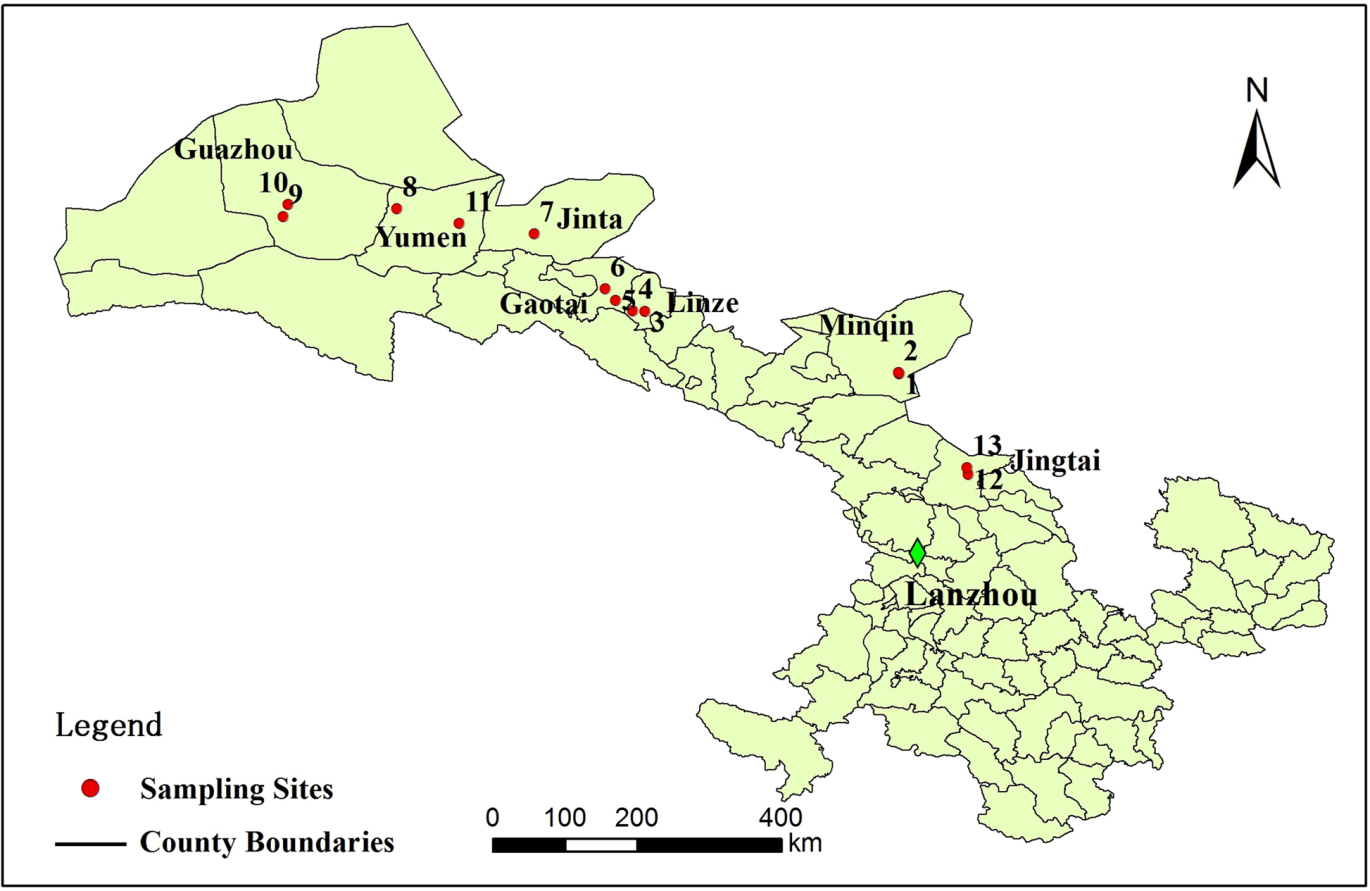
Distribution of the sampling plots among 5 subtypes and 13 genera of saline-alkali soil in arid regions of northwest China

**Fig. 2:**
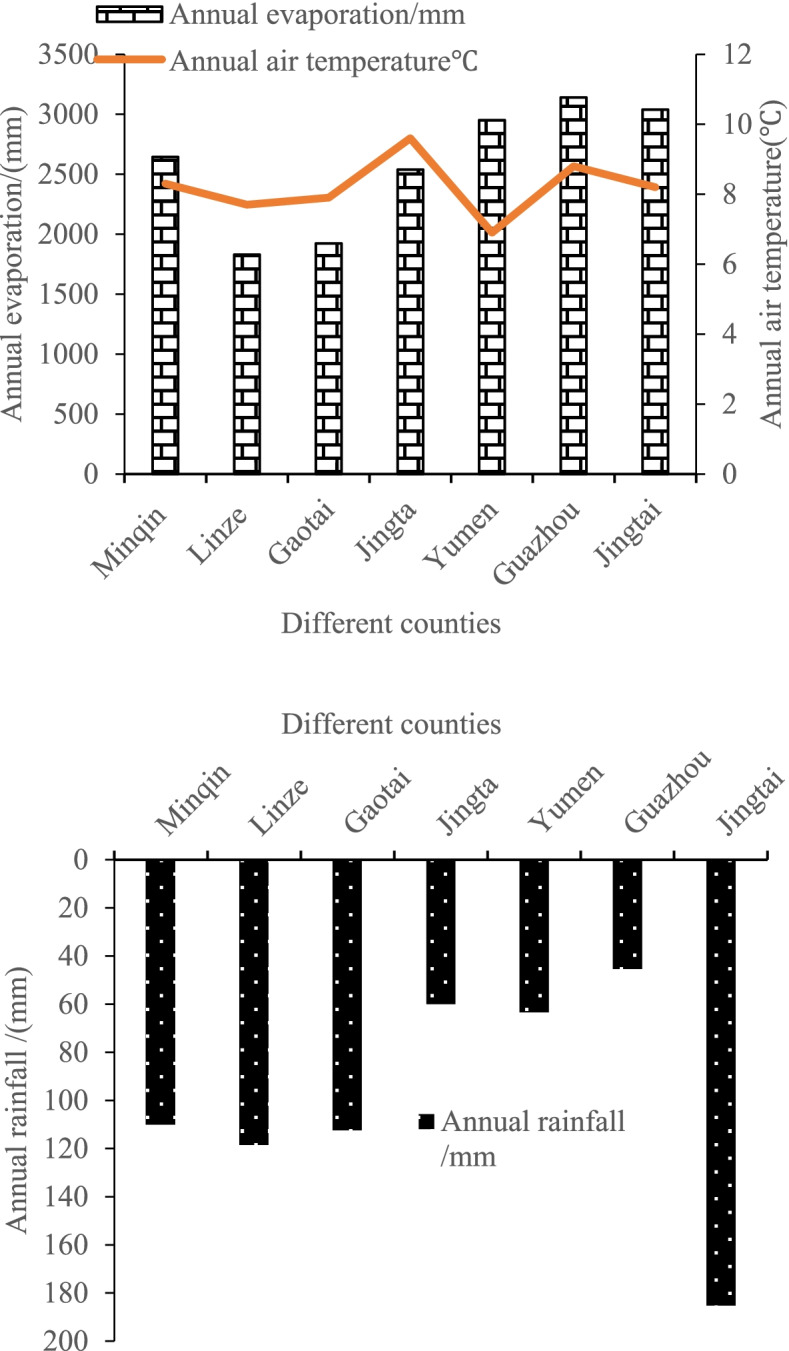
Basic meteorology data of different counties in Gansu Province of China

Changes in archaeal community diversity has been reported in respond to different types of saline-alkali soil in Gansu Province [25]. However, edaphic factors associated with different types of saline-alkali soil on bacterial diversity and composition have not been sufficiently explored. In this study, we collected 39 soil samples in Gansu Province and sequenced on an high throughput Illumina MiSeq sequencing platform to investigate how bacterial diversity and composition has been affected by types of salinity and sodicity. The aim was to identify key factors in shaping bacterial communities among different types of saline-alkali soil in Gansu Province, which is essential for gaining insight into the biological mechanisms of saline adaption (Figs. 1 and 2).

## Results

### Soil biochemical characteristics

There were significant differences (*P* < 0.01) in pH, SOM, TS, SO_4_^2−^, Cl^−^, UA, APA, and DHA among soil samples collected from 13 sites (Table 1). TS contents ranged from 3.81 to 24.35%, which far exceeded the thresholds of 0.2% [9], indicating a high hypersaline environment. DS.ST showed the highest SOM (14.56 g/kg), TS (24.35%). DS.CST had the highest SO_4_^2−^, Cl^−^, DHA and the lowest APA. OS.CT had the highest UA and the lowest SOM and DHA. The lowest TS (3.81%) and UA (0.05 mg/g) were found in MS.SCT. SO_4_^2−^ was the lowest for OS.ST, while MS.ST had the highest APA and the lowest Cl^−^.

**Table 1:** Soil chemical and biological properties of different plots in 5 subtypes and 13 genera of saline-alkali soil in arid regions of northwest China

Treatment groups	pH value	Organic matter content /g/kg	Total salt content /%	Sulfate ion content /%	Chloride ion content /%	Urease mg/g	Alkaline phosphatase mg/g	Dehydrogenase mg/g
MS.CT	8.56	8.27	6.03	1.09	0.38	0.23	0.08	5.51
MS.SCT	8.44	13.61	3.81	1.13	0.44	0.05	0.02	2.04
MS.CST	8.98	10.02	6.07	1.09	0.75	0.36	0.04	1.97
MS.ST.	8.79	9.25	4.09	1.01	0.18	0.16	0.10	4.80
OS.CT	8.98	4.36	11.63	1.06	0.85	0.84	0.02	0.34
OS.SCT	8.37	5.54	15.27	1.31	0.41	0.11	0.02	1.37
OS.CST	8.92	10.76	9.40	1.41	0.63	0.06	0.02	1.87
OS.ST	8.98	5.95	5.45	0.35	0.22	0.22	0.02	5.49
BS.CT	8.85	9.30	4.62	0.77	0.42	0.11	0.02	2.78
AS.Mg.S	9.07	11.32	5.17	1.07	0.57	0.10	0.02	0.36
DS.SCT	8.13	10.59	5.63	1.50	0.82	0.28	0.03	1.23
DS.CST	8.83	7.56	13.96	1.91	1.49	0.33	0.01	7.26
DS.ST	8.36	14.56	24.35	0.69	0.35	0.45	0.02	3.83
*s.e.m.*	0.04^**^	0.05^**^	0.07^**^	0.03^**^	0.06^**^	0.09^**^	0.01^**^	1.06^**^

*Notes*: MS, OS, BS, AS, and DS represent meadow solonchaks, orthic solonchaks, bog solonchaks, alkalized solonchaks, and dry solonchaks, respectively. CT, SCT, CST, ST, Mg.S represent chloride-type, sulfate-chloride-type, chloride-sulfate-type, sulphate-type, and magnesium solonetz, respectively. ^**^P < 0.01; ^*^P < 0.05

### Alpha diversity patterns

Total effective sequences in all soil samples ranged from 47,376 to 64,521 (Table 2), with 97% coverage indicating that the sequence was sufficient. Analysis of the Chao and ACE indices and Shannon and Simpson indices showed that OS.SCT had the lowest species richness and diversity.

**Table 2:** MiSeq sequencing results and diversity indices for 5 subtypes and 13 genera of saline-alkali soil in arid regions of northwest China

Treatment groups	Effective Tags	OTUs	Chao1	ACE	Coverage %	Simpson	Shannon
MS.CT	61,711	3123	3229.32	3323.79	97.4	0.988	8.69
MS.SCT	57,043	2507	2803.08	2814.55	97.7	0.987	8.17
MS.CST	56,870	2843	3021.27	3201.71	97.4	0.987	8.37
MS.ST.	53,843	3254	3281.32	3368.13	97.5	0.994	9.18
OS.CT	64,521	3148	3202.21	3249.52	97.4	0.983	8.37
OS.SCT	59,089	1994	2284.12	2417.71	98.0	0.975	7.32
OS.CST	59,821	2880	2900.17	3023.69	97.7	0.993	8.88
OS.ST	63,853	2978	3260.80	3377.49	97.4	0.994	9.08
BS.CT	57,067	3086	3417.98	3434.16	97.1	0.988	8.53
AS.Mg.S	55,360	2865	3037.54	3177.13	97.4	0.984	8.30
DS.SCT	47,376	2353	3047.13	2927.60	97.6	0.981	7.82
DS.CST	58,687	2853	3047.40	3117.45	97.5	0.984	8.19
DS.ST	53,200	2268	2369.02	2430.74	98.2	0.987	8.13
*s.e.m.*	0.208	75.038^**^	103.42^**^	130.54^**^	0.004	0.005^*^	0.392^**^

*Notes*: MS, OS, BS, AS, and DS represent meadow solonchaks, orthic solonchaks, bog solonchaks, alkalized solonchaks, and dry solonchaks, respectively. CT, SCT, CST, ST, Mg.S represent chloride-type, sulfate-chloride-type, chloride-sulfate-type, sulphate-type, and magnesium solonetz, respectively. ^**^P < 0.01; ^*^P < 0.05

### Change in bacterial community compositions in soil samples

**Fig. 3:**
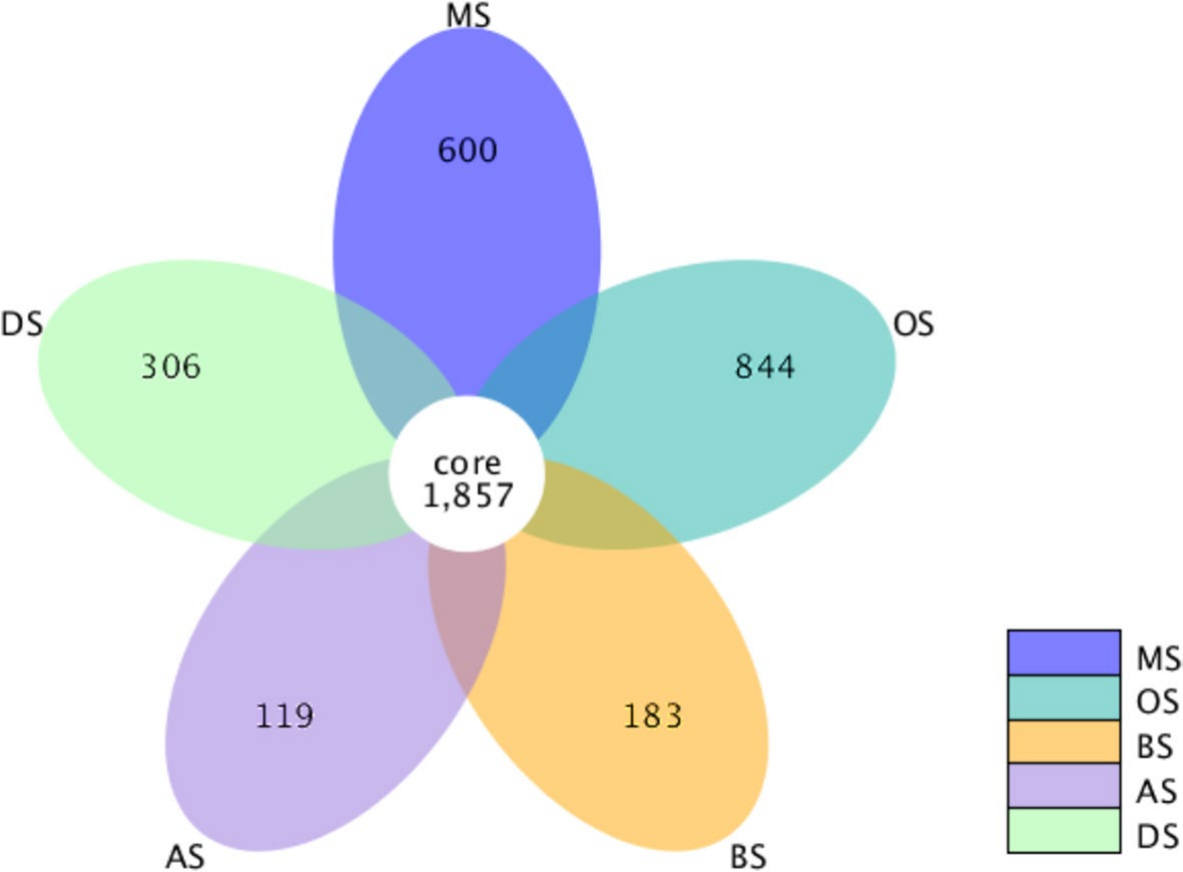
Flower diagram showing the shared bacterial OTUs in all soil samples. *Notes*: MS, OS, BS, AS, and DS represent meadow solonchaks, orthic solonchaks, bog solonchaks, alkalized solonchaks, and dry solonchaks, respectively

Flower diagram revealed the total observed OTUs in soil samples (Fig. 3), and 1857 OTUs were common to all soil samples. Moreover, the distribution of sequences also demonstrated that each subtype had its own microbial population.

**Fig. 4:**
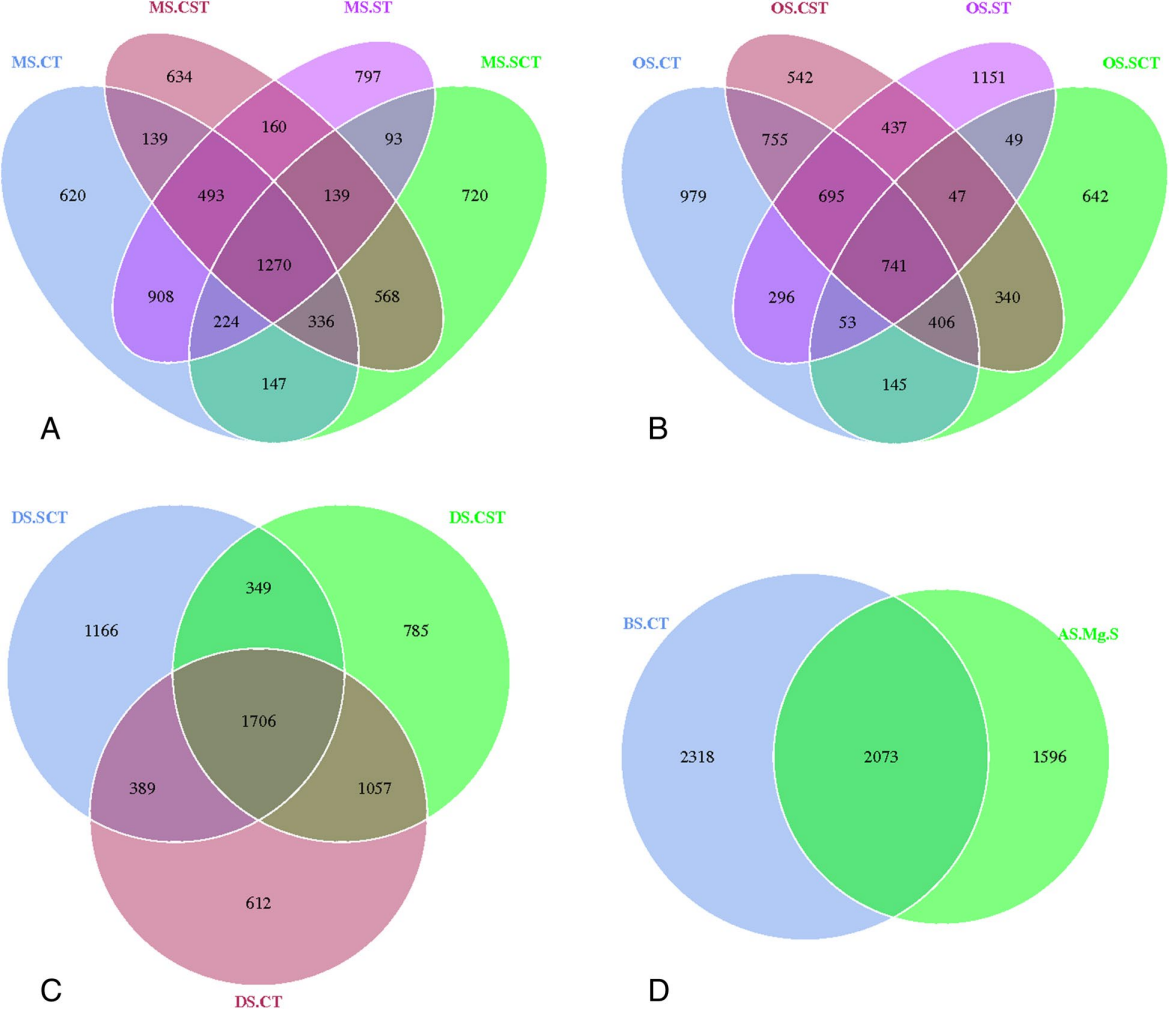
Venn graph of the numbers of shared and unique operational taxonomic units (OTUs) among 5 subtypes and 13 genera of saline-alkali soil in arid regions of northwest China The overlapping part represents the shared OTUs; non-overlapping part represents the specific OTUs of the given genera; and the number means the corresponding amount of OTUs. *Notes*: MS, OS, BS, AS, and DS represent meadow solonchaks, orthic solonchaks, bog solonchaks, alkalized solonchaks, and dry solonchaks, respectively. CT, SCT, CST, ST, Mg.S represent chloride-type, sulfate-chloride-type, chloride-sulfate-type, sulphate-type, and magnesium solonetz, respectively

Venn diagrams revealed the total observed OTUs in soil samples (Fig. 4). The numbers of unique OTUs for MS.CT, MS.CST, MS.SCT, and MS. ST was 620, 634, 720 and 780, respectively. The number of shared OTUs for 4 soil genera was 1270, accounting for 17.52% of all observed OTUs (Fig. 4A). The number of specific OTUs in OS.CT, OS.CST, OS.SCT, and OS.ST were 979, 542, 642 and 1151, respectively. The total number of OTUs for 4 soil genera were 741(Fig. 4B), accounting for 10.18% of the total OTUs. The number of specific OTUs in DS.CT, DS.CST, and DS.SCT was 612, 785 and 1166, respectively. The total number of OTUs for 3 soil genera was 1706 (Fig. 4C), accounting for 28.13% of the total OTUs. The number of specific OTUs in BS.CT and AS.MgS was 2318 and 1596, respectively. The total OTUs for bacteria was 2073 (Fig. 4D), accounting for 34.63% of the total OTUs.

**Fig. 5:**
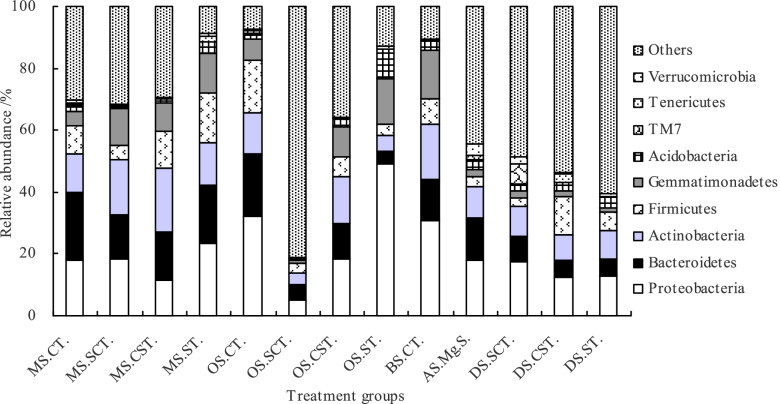
Relative abundance of soil bacterial community at phylum level among 5 subtypes and 13 genera of saline-alkali soil in arid regions of northwest China. *Notes*: MS, OS, BS, AS, and DS represent meadow solonchaks, orthic solonchaks, bog solonchaks, alkalized solonchaks, and dry solonchaks, respectively. CT, SCT, CST, ST, Mg.S represent chloride-type, sulfate-chloride-type, chloride-sulfate-type, sulphate-type, and magnesium solonetz, respectively

The main bacterial phyla included *Proteobacteria*, *Bacteroidetes*, *Actinobacteria*, *Firmicutes*, *Gemmatimonadetes*, *Acidobacteria*, *TM7*, *Tenericutes*, and *Verrucomicrobia* (Fig. 5). The relative abundances of these phyla together made up an average of 65.0% for all bacteria. ANOVA revealed that except for *Firmicutes* and *Tenericutes*, other bacterial phyla showed significant differences in all samples (*P* < 0.01 or *P* < 0.05). *Proteobacteria* was the most abundant in MS.ST, OS.ST, OS.CT, BS.CT, DS.SCT, DS.CST, and DS.ST.

**Fig. 6:**
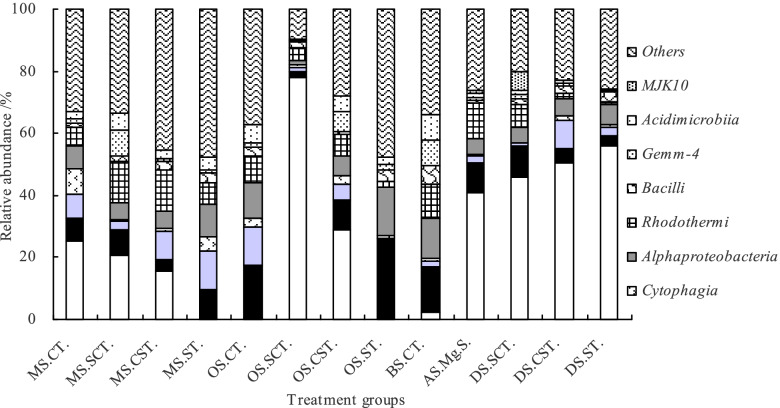
Relative abundance of soil bacterial community at class level among 5 subtypes and 13 genera of saline-alkali soil in arid regions of northwest China. *Notes*: MS, OS, BS, AS, and DS represent meadow solonchaks, orthic solonchaks, bog solonchaks, alkalized solonchaks, and dry solonchaks, respectively. CT, SCT, CST, ST, Mg.S represent chloride-type, sulfate-chloride-type, chloride-sulfate-type, sulphate-type, and magnesium solonetz, respectively

Average relative abundance of the top 10 microbial dominant classes accounted for 68.44% of all bacterial classes (Fig. 6). There were significant differences in *Halobacteria* (*s.e.m* = 0.156^**^), *Gammaproteobacteria* (*s.e.m* = 0.035^**^), *Clostridia* (*s.e.m* = 0.037^**^), *Cytophagia* (*s.e.m* = 0.021^*^), *Alphaproteobacteria* (*s.e.m* = 0.029^**^), *Rhodothermi* (*s.e.m* = 0.025^**^), *Gemm-4* (*s.e.m* = 0.014^**^), *Acidimicrobiia* (*s.e.m* = 0.013^**^), and *MJK10* (*s.e.m* = 0.009^**^) except for *Bacilli* in soil samples.

### Relationship between bacterial community compositions and edaphic factors

SOM, pH, TS, SO_4_^2−^, Cl^−^, UA, DHA, and APA were closely correlated with the abundance of the dominant bacterial phyla (Table 3). The abundance of *Proteobacteria* (*r* = − 0.319, *p* = 0.047) was correlated negatively with SOM. The abundance of *Proteobacteria* (*r* = 0.386, *p* = 0.015), *Bacteroidetes* (*r* = 0.456, *p*<0.01), *Actinobacteria* (*r* = 0.492, *p*<0.01), *Firmicutes* (*r* = 0.332, *p* = 0.039), and *Gemmatimonadetes* (r = 0.499, *p*<0.01) showed positive relationship with pH. The abundance of *Proteobacteria* (*r* = − 0.343, *p* = 0.032), *Bacteroidetes* (*r* = − 0.407, *p* = 0.01), *Actinobacteria* (*r* = − 0.325, *p* = 0.043), and *Gemmatimonadetes* (r = − 0.561, *p*<0.01) had a negative correlation with TS.

**Table 3:** Pearson’s correlation (r) and significance (p) values between the abundances of the dominant abundant bacterial phyla and the soil variables

Taxonomic group	Soil organic matter content		pH		Soil total salt content		Sulfate anion content		Chloride anion content		Urease		Dehydrogenase		Alkaline phosphatase	
	*r*	*p*	*r*	*p*	*r*	*p*	*r*	*p*	*r*	*p*	*r*	*p*	*r*	*p*	*r*	*p*
Proteobacteria	−0.319^*^	0.047	0.386^*^	0.015	−0.343^*^	0.032	−0.560^**^	0.000	−0.260	0.110	0.192	0.242	0.060	0.717	−0.064	0.700
Bacteroidetes	− 0.033	0.842	0.456^**^	0.004	− 0.407^*^	0.010	− 0.051	0.758	− 0.118	0.475	0.083	0.617	−0.105	0.523	0.616^**^	0.000
Actinobacteria	0.293	0.070	0.492^**^	0.001	− 0.325^*^	0.043	− 0.030	0.857	0.000	1.000	0.069	0.677	−0.154	0.349	0.058	0.727
Firmicutes	−0.231	0.157	0.332^*^	0.039	−0.002	0.992	0.051	0.759	0.168	0.305	0.483^**^	0.002	0.114	0.489	0.251	0.122
Gemmatimonadetes	−0.051	0.760	0.499^**^	0.001	−0.561^**^	0.000	−0.451^**^	0.004	−0.387^*^	0.015	−0.218	0.183	0.068	0.682	0.168	0.306
Acidobacteria	−0.151	0.360	0.109	0.509	−0.104	0.530	−0.495^**^	0.001	−0.288	0.075	−0.023	0.888	0.354^*^	0.027	−0.060	0.718
TM7	0.135	0.412	−0.228	0.162	−0.183	0.264	0.265	0.103	0.166	0.311	0.041	0.804	−0.191	0.243	−0.042	0.800
Tenericutes	−0.172	0.295	−0.039	0.812	0.101	0.541	0.317^*^	0.049	0.346^*^	0.031	0.310	0.054	0.247	0.129	0.118	0.474
Verrucomicrobia	0.175	0.287	−0.046	0.783	−0.117	0.479	0.024	0.884	0.034	0.836	−0.027	0.871	−0.191	0.244	−0.082	0.621

^**^P < 0.01; ^*^P < 0.05

The abundance of *Proteobacteria* (*r* = − 0.560, *p*<0.01), *Gemmatimonadetes* (*r* = − 0.451, *p*<0.01), and *Acidobacteria* (*r* = − 0.495, *p*<0.01) were correlated negatively with SO_4_^2−^, then *Gemmatimonadetes* (r = − 0.387, *p* = 0.015) had a negative correlation with Cl^−^, while *Tenericutes* (r = 0.317, *p* = 0.049; r = 0.346, *p* = 0.031, respectively) showed positive relationship with SO_4_^2−^ and Cl^−^. Additionally, there was a significant positive correlation between *Firmicutes* (r = 0.483, *p*<0.01) and UA, *Acidobacteria* (r = 0.354, *p* = 0.027) and DHA, *Bacteroidetes* (r = 0.616, *p*<0.01) and APA.

**Fig. 7:**
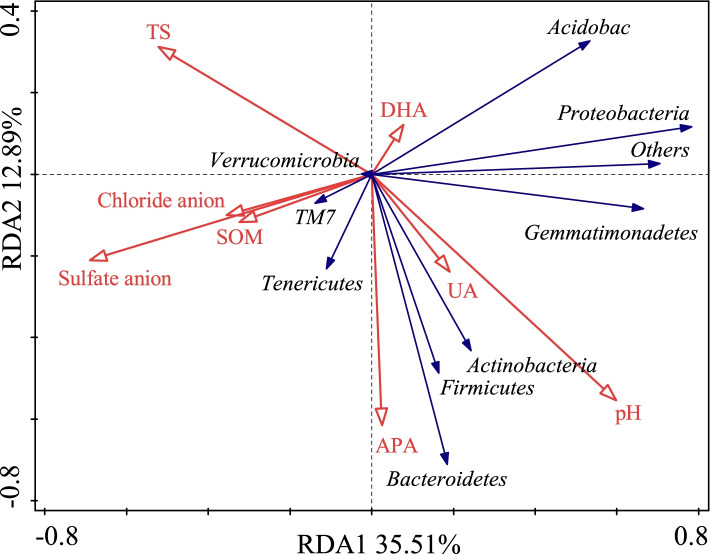
Redundancy analysis (RDA) of the relationships between microbial composition and edaphic factors of 5 subtypes and 13 genera of saline-alkali soil in arid regions of northwest China. *Notes*: TS, DHA, UA, and APA represent total salt content, dehydrogenase, urease, and alkaline phosphatase, respectively

The two redundancy analysis (RDA) axes explained 48.4% of the variation between the soil bacterial communities (Fig. 7). The distinctions of bacterial community structure among 5 subtypes and 13 genera of soil were also supported by the redundancy analysis (RDA). Soil SO_4_^2−^, pH value, APA, TS, SOM, UA, and DHA had significant effects on bacterial community compositions (*P* = 0.001 by the Monte Carlo permutation test) (Table 4).

**Table 4:** Simple term effects of environmental variables based on Monte Carlo permutation tests from the redundancy analysis

Variable	Forward selection			
	Contribution %	Explains %	pseudo-F	P
Sulfate anion content	17.7	30.9	7.9	0.002
pH value	11.7	20.4	5.9	0.006
Alkaline phosphatase	6.8	11.8	4.0	0.016
Soil total salt content	6.6	11.5	3.6	0.018
Soil organic matter content	6.2	10.8	4.0	0.012
Urease	3.8	6.6	2.5	0.042
Dehydrogenase	3.9	6.8	2.8	0.038
Chloride anion content	0.8	1.4	0.5	0.71

## Discussion

### Bacterial community diversity and structure

Bacteria are the most abundant and diverse group of soil microorganisms which play multiple important key roles in soil [23]. We collected 13 saline-alkali soils (5 subtypes and 13 genera) to determine their bacterial diversity and abundance. It was found that the bacterial communities differed greatly among 13 soil samples, and the richness estimators (Ace and Chao 1) and the diversity indices (Shannon and Simpson) revealed a least diversity in bacterial community in OS.SCT.

*Proteobacteria*, *Bacteroidetes*, *Actinobacteria*, *Firmicutes*, and *Gemmatimonadetes* accounted for a large proportion (17.81–89.26%) among different treatments, which was consistent with previous study of bacterial community analysis in saline-alkali soil [24, 38]. *Proteobacteria* was a ubiquitous and common group in soil [7] and was reported as “salinity related” [42]. Our results further confirmed this finding. *Firmicutes* which was considered special indicators specifically in high salt soil [38], occurred at a level of 2.51-16.67% in all samples in this study, but *Firmicutes* was absent in various hyper saline environments in previous studies [8, 29]. *Gemmatimonadetes* and *Bacteroidetes* are also important participant in biogeochemical transformations in soils under salinity [14]. The *Actinobacteria* and *Acidobacteria* have been depicted as common inhabitants of all soils [18, 44]. Furthermore, *Actinomycetes* can significantly inhibit the activities of some plant pathogenic fungi, and can also promote the germination of seeds and the growth of plant roots [20]. *Halobacteria* was the dominant bacterial class in all soils except for MS.ST, OS.CT, OS.ST, and BS.CT. Members of class haloarchaea are obligately halophilic, predominantly aerobic heterotrophs that consistently contribute to the biogeochemical cycling of carbon and nitrogen in saline soils [1, 37].

### Effects of soil properties on the dominant bacterial phyla and bacterial community structure

Microorganisms are particularly sensitive to environmental change [27]. The Monte Carlo permutation test showed that pH, TS, SO_4_^2−^, and UA were significantly correlated with change in bacterial community composition in 13 saline soils. The soil pH account for 32.1% of variance in bacterial community, and acted as the most important driver in bacterial community, in consistent with other finding [11, 20, 34]. In addition, TS was an important factor affecting change in microbial community composition in saline soils in this study, as also reported by Rath et al. [30]. Change in bacterial community is mainly affected by SO_4_^2-^, a dominant anion in saline-alkali soils of Gansu Province. Enzymatic activity is a sensitive parameter and is influenced by a number of natural and anthropogenic factors [26]. Our soil samples showed a positive relationship between bacterial abundance and enzyme especially UA, suggesting soil halophiles enzymes require salt for maintaining enzyme stability and activity [21].

## Conclusions

Our results demonstrated that *Proteobacteria*, *Bacteroidetes*, *Actinobacteria*, *Firmicutes*, and *Gemmatimonadetes* were the main indicator species reflecting changes of the main microbial groups and the richness and diversity of bacterial communities were the lowest in sulfate-chloride-type orthic solonchaks. The sulfate anion as a key factor drives the composition of the bacterial community among 5 subtypes and 13 genera of saline-alkali soil of Gansu Province.

## Material and methods

### Site description

The study area located in Gansu Province (37°17′-42°48′N, 92°12′-103°48′E), Northwest China, which belonged to a transitional zone between arid and semi-arid regions. It was affected by continental climate and Tibetan Plateau climate with annual mean temperature of 5-10 °C, annual mean evaporation of 1448.4 mm. The altitude is 1100-1500 m above sea level; frost-free days is 160 to 230 days; the sunshine duration is more than 3000-4000 h/a; annual total solar radiation is 120 × 4, 186.8-150 × 4, 186.8 J/cm^2^ [25].

Saline soils of this region were divided into five subtypes (meadow solonchaks, orthic solonchaks, bog solonchaks, alkalinzed solonchaks, and dry solonchaks, shorting for MS, OS, BS, AS, DS, respectively.). Meadow solonchaks and orthic solonchaks were further divided into four genera, including sulphate type (ST), chloride-sulphate type (CST), sulphate-chloride type (SCT), and chloride type (CT). Bog solonchaks and alkalinzed solonchaks were divided into only one genera, namely bog solonchaks (BS) and magnesium alkalinzed solonchaks (Mg.S), respectively. Dry solonchaks were divided into three genera, including sulphate type (ST), chloride-sulphate type (CST), sulphate-chloride type (SCT) [25]. Most plants in this region are halophyte species. The vegetation pattern consists of *Achnatherum splendens* (Trin.)*, Nitraria tangutorum* Bobr.*, Phragmites australis* (Cav.), *Tamarix ramosissima* L., *Glycyrrhiza uralensis* Fisch., *Alhagi sparsifolia* Shap., *Leymus secalinus* (Georgi) Tzvel, *Suaeda glauca* (Bunge). The vegetation coverage is 0.76, 24.27, 38.50, 19.10, 53.25, 31.37, 37.29, 37.87, 43.68, 29.40, 48.40, 43.67, and 35.32% in MS.CT, MS.SCT, MS.CST, MS.ST, OS.CT, OS.SCT, OS.CST, OS.ST, BS.CT, AS.MgS, DS.SCT, DS.CST, and DS.ST, respectively.

### Site selection and soil sampling

The thirteen study sites and basic meteorology data of different counties were shown in Figs. 1 and 2, respectively. At each study site, three sample lines were selected with about 120 degree (angle) between adjacent lines. Five soil cores between adjacent cores spacing 5 m, at a depth of 0–10 cm, were taken from each lines and combined to obtain about 100 g of soil in July, 2015, for a total of 39 samples. Samples were mixed thoroughly, placed in an ice box and brought back to the laboratory immediately. Soil sample was divided into two subsamples: one was air dried, 2 mm sieved, then chemically and physically analysed, while the second one was stored at − 80 °C and later processed for high-throughput sequencing.

### Edaphic factors characteristics

Soil organic matter (SOM) was determined using K_2_Cr_2_O_7_-H_2_SO_4_ digestion [4]. Potentiometric titration was used to determine Cl^−^ and SO_4_^2−^ with air-dried soil (soil: water, 1:5). Total salt content (TS) was measured in soil suspensions (soil: water = 1:5) with a conductance instrument, and pH was determined with air-dried soil (soil: water, 1:5) by pH meter (PHS-3C, REX, Shanghai). The enzymatic activities of urease (UA), alkaline phosphatase (APA), and dehydrogenase (DHA) were determined according to the methods described by Guan [16].

### Soil DNA extraction

DNA was extracted by cetyltrimethyammonium ammonium bromide (CTAB) method. The final DNA concentration and purification were examined using a Nano Drop spectrophotometer (Nano Drop Technologies, Wilmington, DE, USA), and DNA quality was checked by 1% agarose gel electrophoresis.

### Amplification and sequencing of 16S rRNA genes

Next generation sequencing was performed in Novogene, Inc. (Beijing, China). The primer pair 515F (5′-GTGCCAGCMGCCGCGGTAA-3′) and 806R (5′-GGACTACHVGGGTWTCTAAT-3′) was used to amplify the V4 region of bacterial 16S rRNA gene [6]. PCR amplification was carried out with Phusion® High-Fidelity PCR Master Mix (New England Biolabs). PCR products were purified with Qiagen Gel Extraction Kit (Qiagen, Germany). The libraries were constructed with the TruSeq® DNA PCR-Free Sample Preparation Kit (Illumina, USA). The library quality was quantified by Qubit and quantitative real-time PCR.

Paired-end reads were merged using FLASH [22]. Raw high-throughput sequencing data was processed using QIIME toolkit and the UPARSE pipeline [5, 10]. After filtering DNA sequences using quality files, the remaining sequences were trimmed to remove barcodes and forward primers. The low quality sequences were excluded. The sequencing data were pre-treated to remove the chimeras from the datasets. The sequencing data were pre-treated to remove the chimeras from the datasets. Sequencing results of samples were defined as operational taxonomic units (OTUs) at 97% identity threshold. Species annotation was performed using the GreenGene Database based on the Ribosomal Data Project (RDP) database [10]. All sequencing data were deposited in the NCBI Sequence Read Archive database (BioProject ID PRJNA707428, study accession number SRX10280109 to SRX10280147).

## Statistical analysis

Analysis of variance was performed for each measured soil variable, and variance was compared between groups by Duncan’s test (*P* < 0.05). Chao1, ACE, Shannon and Simpson indexes were calculated by QIIME with normalized data [28]. The flower and Venn diagrams were performed using R software (version: 2.15.3). Pearson’s correlation (*r*) and significance (*p*) values were performed using SPSS version 20. Redundancy analysis (RDA) was used to examined the relationships between the environmental factors and bacterial community structure with CANOCO 5.0 for Windows (Monte Carlo permutation test, *P* = 0.001; Microcomputer Power, USA).

## Data Availability

The genome sequences can be accessed after 2021-04-01 at https://www.ncbi.nlm. nih.gov/bioproject/browse using Bioproject PRJNA707428. Until then, the sequences are available from the corresponding author upon reasonable request.

## Abbreviations

MS: Meadow solonchaks
OS: Orthic solonchaks
BS: Bog solonchaks
AS: Alkalized solonchaks
DS: Dry solonchaks
CT: Chloride-type
SCT: Sulfate-chloride-type
CST: Chloride-sulfate-type
ST: Sulphate-type
Mg.S: Magnesium solonetz
